# Supplementary material for: Asymmetric Synthesis of Quaternary Hydantoins via a Palladium-Catalyzed Aza-Heck Cyclization
Source: J Am Chem Soc. 2025 Nov 14;147(49):44692–8. doi: 10.1021/jacs.5c16022 (PMC12703750; doi:10.1021/jacs.5c16022)
Supplement: Supplementary file 2 [file ja5c16022_si_002.zip › All NMR FID Files/S45/S45_CarbonNMR/1/pdata/1/email_MLM03166C1-CAPT_1_1.pdf]

C13APT1024 CDCl3 /opt/nmrdata mlmurr 17

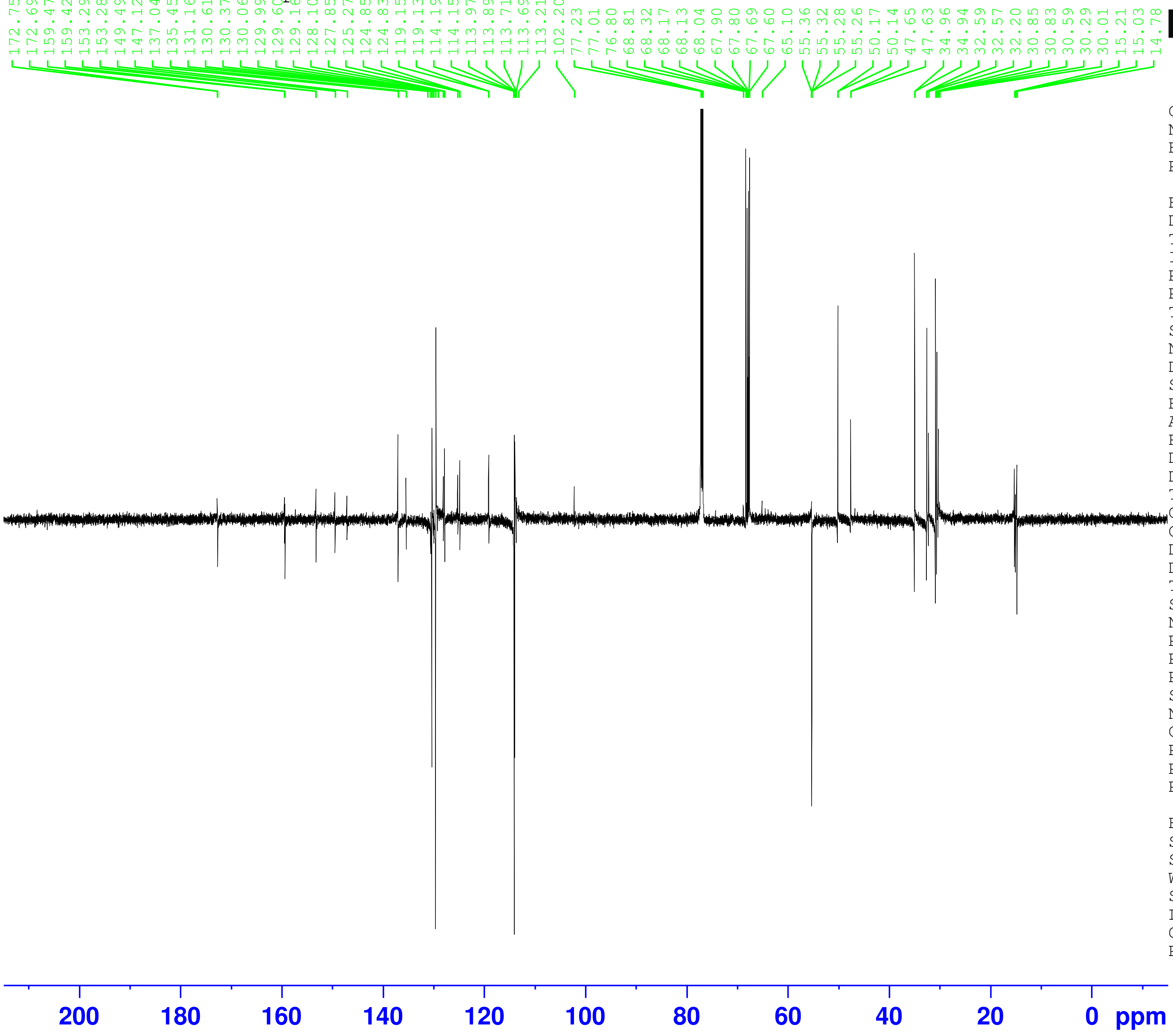

BRUKER

Current Data Parameters

NAME	MLM03166C1-CAPT	
EXPNO	1	
PROCNO	1	

F2 - Acquisition Parameters

Date_	20250214	
Time	20.44	h
INSTRUM	spect	
PROBHD	Z168773_0053 (	
PULPROG	jmod.acqt0	
TD	65536	
SOLVENT	CDCl3	
NS	1024	
DS	4	
SWH	34722.223	Hz
FIDRES	1.059638	Hz
AQ	0.9437184	sec
RG	2050	
DW	14.400	usec
DE	18.88	usec
TE	300.0	K
CNST2	145.0000000	
CNST11	1.0000000	
D1	5.00000000	sec
D20	0.00689655	sec
TD0	1	
SFO1	150.9656791	MHz
NUC1	13C	
P1	10.00	usec
P2	20.00	usec
PLW1	88.38300323	W
SFO2	600.3224013	MHz
NUC2	1H	
CPDPRG[2	waltz16	
PCPD2	80.00	usec
PLW2	21.85499954	W
PLW12	0.48474309	W

F2 - Processing parameters

SI	32768	
SF	150.9505840	MHz
WDW	EM	
SSB	0	
LB	1.00	Hz
GB	0	
PC	1.40	
